# GLUT4 On the move

**DOI:** 10.1042/BCJ20210073

**Published:** 2022-02-11

**Authors:** Daniel J. Fazakerley, Francoise Koumanov, Geoffrey D. Holman

**Affiliations:** 1 Metabolic Research Laboratories, Wellcome-Medical Research Council Institute of Metabolic Science, University of Cambridge, Cambridge CB2 0QQ, U.K.; 2 Department for Health, Centre for Nutrition, Exercise, and Metabolism, University of Bath, Bath, Somerset BA2 7AY, U.K.; 3 Department of Biology and Biochemistry, University of Bath, Bath, Somerset BA2 7AY, U.K.

**Keywords:** glucose transport, GLUT4, insulin, membrane traffic, membrane trafficking kinetics, signal transduction

## Abstract

Insulin rapidly stimulates GLUT4 translocation and glucose transport in fat and muscle cells. Signals from the occupied insulin receptor are translated into downstream signalling changes in serine/threonine kinases within timescales of seconds, and this is followed by delivery and accumulation of the glucose transporter GLUT4 at the plasma membrane. Kinetic studies have led to realisation that there are distinct phases of this stimulation by insulin. There is a rapid initial burst of GLUT4 delivered to the cell surface from a subcellular reservoir compartment and this is followed by a steady-state level of continuing stimulation in which GLUT4 recycles through a large itinerary of subcellular locations. Here, we provide an overview of the phases of insulin stimulation of GLUT4 translocation and the molecules that are currently considered to activate these trafficking steps. Furthermore, we suggest how use of new experimental approaches together with phospho-proteomic data may help to further identify mechanisms for activation of these trafficking processes.

## Introduction

In the 100th year of the discovery of insulin we reflect on a famous cartoon of the 1970s in which ‘Chuck’ describes the black box theory of insulin action and the following sequence: insulin binds to its receptor; something happens; cellular effects [1]. Since that time, some 45 years later, the black box ‘something happens’ could be replaced by ‘lots of things happen, but not all of them happen quickly’. So, what can we now learn from the available catalogue of necessary proteins, their subcellular compartments, and the kinetics of the insulin response?

Pioneering cell biology studies [2,3] revealed that insulin stimulates glucose transport into fat and muscle cells through translocation of the glucose transporter from intracellular stores to the plasma membrane (PM). Since the discovery of GLUT4 in the late 1980s [4–6], studies have shed light on the GLUT4 complex intracellular trafficking itinerary that effectively excludes GLUT4 from the PM in the absence of insulin and provides a large reservoir of GLUT4 that can be readily mobilised in response to insulin. Very early in these studies it became evident that insulin stimulation of glucose uptake and GLUT4 translocation is a very fast process, and that maximum glucose uptake is achieved within 10 min of insulin binding to its receptor (Table 1).

**Table 1.** Key signalling and trafficking components in GLUT4 translocation. See key at foot of Table p5

process	key effectors	insulin regulated	PI3K dependent	Akt dependent	time scale of activation including phosphorylation (full activation unless otherwise indicated) **Phosphorylation times from ref**. [36]
Overall process					
Glucose transport		Yes	Yes	Yes	½ time = 5–10 min
Signalling					
Formation of the IR–IRS–PI3K complex*	Insulin receptor, IRS, PI3K [36,106,107]	Yes			<1 min **InsR and IRS1/2 pTyr in 15 s**
IP3 synthesis at the PM*	PI3K Class 1a [13,33,108–112]	Yes	Yes		<1 min ½ time — 2.4 s [33]
Akt activation*	Akt, PDK1, mTORC2 [16,36,107]	Yes	Yes **Yes**	Yes **Yes**	Akt translocation <1 min; Akt phosphorylation <1 min **(Akt2 pThr309 in 15 s; pSer474 in 30 s)**; Akt activation (substrate phosphorylation) <1 min **(30 s)** [36] **mTORC2 multiple phosphosites, full activation within 5 min**
Actin remodelling	EHD1* [113], EHBP1 [113], EHD2 [106,114]	Yes	**Yes**	**Yes**	pTyr453 EHD2 within 5 min [106] **EHD1 — pSer456 in 20 min** **EHBP1 — pSer1061 in 30 s** **EHD2 — pSer451 in 15 s — only PI3K-dependent**
	CAP, TC10* [74,115,116]	Yes	No **No**	No **No**	**CAP — multiple Ser/Thr phosphosites some, as Ser287, are activated in 5 min and are PI3K- and Akt-dependent perhaps via a feedback loop**. **TC10 — pSer9 in 1 min** No time course of GTP loading reported
	Tropomodulin3 [117]	Yes	Yes **No**	Yes **No**	**pSer71 in 2–5 min**
	Tropomyosin3.1 [118]	Yes	N.R. **No**	N.R. **No**	**pSer51 in 20–60 min**
	CLASP2 (microtubules, membrane ruffles) [119,120]	Yes	No	No	**Several Ser phosphosites in 2–60 min** *Serine phosphorylation* via *MAPK* [120]
	*Rac1* [121,122]	*Yes*	*Yes*	*No*	**NPD**
	Rab13, MICAL-L2, Actinin4* [123]	*Yes*	*Yes* **Yes**	*No* **No**	*2–5 min time course of GTP loading* **Rab13 pTyr5 in 15 s pSer178 in 10 min** **MICAL-L2-pSer143 in 15 s**; **Actinin4 — pSer424 in 5 min (transient)**
GLUT4 vesicle trafficking					
Translocation of GSVs to PM and tethering of GSV at PM	TBC1D4* [34,35,58,100,124–127]	Yes	Yes **Yes**	Yes **Yes**	**TBC1D4 phosphorylation 15–30 s on different Ser/Thr residues** Interaction with 14-3-3 [58,125,127], IRAP [100,128]
	Rab10 (GTP loading) [67]	No	N.R. **Yes**	N.R. **No**^†^	**pTyr6 in 15 s; Slower pThr73 in 10 min**
	DENND4c (Rab GEF) [92]	No (GEF activity)	N.R. **Yes**	N.R. **Yes**	pSer1043, pSer1096 and pSer1321 insulin-sensitive phosphorylation. **Multiple phosphosites; pSer 1289 fastest in 2–5 min**
	Sec16 [63,129,130]	Yes	N.R. **Yes**	Yes **No**	**Sec16a multiple phosphosites. Fastest pSer2311 in 2 min** **Sec16b pSer182/pSer185 Moderate activation 30 s**
	*TBC1D1* [127,131–133]	*Yes*	*Yes*	*Yes*	**5 phosphosites detected, only pSer231 is insulin sensitive, but not above threshold value of 0.5**
	*Rab8 and Rab13* [123,134]	*Yes*	*N.R*. **No**^†^	*N.R.* **No**^†^	*2–5 min time course of GTP loading* **Rab8: Transient phosphorylation pSer181/185 30 s to 1 min** **Rab13 see above**
	MyoVA [68,135,123,134]	Yes	Yes **No**	Yes **No**	Akt substrate pSer1650 in 5–10 min **pSer600 and pSer1650 in 10 min**
	Synip [69]	Yes	Yes **No**^†^	Yes **No**^†^	Akt substrate, phosphorylation in 5–10 min **Only pSer12 at 60 min**
	RGC2/AS250 (RalGAP)* [71]	Yes	Yes **Yes**	Yes **Yes**	Akt substrate, phosphorylation within 5 min **Multiple phosphosites. Fastest Thr715 in 15 s + pSer486/696/765 in 30 s**
	RalA [71,75]	Yes	Yes	Yes	<5 min for GTP loading **NISPD**
	Exocyst (Exo70, Exo 84 [77,96,136], Sec6 [137], Sec8 [138])	Yes	No **No**	N.R. **No**	Exo 84 is dependent on transient phosphorylation (2–5 min) of TBK1. **Exo70 pSer 242/245/250 at 60 min; Sec 8 pSer32 in 5–10 min. Exo84 NISPD**
	Myo1C [75,139–141,142]	Yes	Yes **No**^†^	No **No**^†^	Phosphorylation 2–5 min **Small activation at 2 min pSer375**
	CAMKII [141]	Yes	N.R. **No**^†^	No **No**^†^	**Several phosphosites activated in 60 min**
	Rab3(B,D), Noc2 [73]	Yes	No	No	<5 min for GTP loading **NPD** **Rab3GAP1 and Rab3IP are phosphorylated within 30 s**
GSV docking, fusion with PM and dispersal of GLUT4 in the PM	Munc18c* [72,86,90,91,106]	Yes	No **No**	No **No**	pTyr 5 min **pSer588 in 15 s to 2 min**
	Doc2B [143]	Yes	N.R.	N.R.	**NPD**
	Tomosyn1/2* [70,97,144,145]	Yes	Yes **No**	Yes **No**	Akt substrate Ser783[70]. Timing <5 min **pSer724/786/783 all within 15–30 s**
	Syntaxin4* [79,146]	Yes	Yes **Yes**	Yes **Yes**	Stx4 pY [36,106] 30 s for full activation **pTyr251 30 s**; **Multiples phosphosites: Ser208 in 15 s; pSer248 transient 2–5 min but no PI3K or Akt dependence**
	SNAP23* [80,81]	N.R.	N.R. **No**^†^	N.R. **No**^†^	**pThr24 transient 30 s to 2 min**
	Vamp2 [57,78,147]	N.R.	N.R. **Yes**	N.R. **Yes**	**pSer48 in 5–10 min**
	*Complexin-2* [148]	*Yes*	*N.R.* **No**	*No* **No**	**pSer93 in 30 s**
	NSF and αSNAP* [93,95,149,150]	N.R.	N.R. **No**	N.R. **No**	**NSF: pSer207 in 15 s** **αSNAP: Ser24 in 20–60min**
	EFR3 and PI 4-kinase IIIα* [66]	N.R.	N.R. **Yes**	N.R. **Yes**	**EFR3: very low Ser360/363 in 15 s**
GLUT4 internalisation (endocytosis)	Clathrin [98,151]	Yes	N.R. **No**	N.R. **No**	**Heavy chain: pTyr434 in 15 s**
	Dynamin-2* [152–154]	Yes	N.R. **No**	N.R. **No**	**NPD but Dynamin-1 (Dnm) detected with multiple phosphosites — some in 15 s or 30 s**
	CIP4/2 [74] N-WASp, CIP4* [155]	Yes *Yes*	N.R. **No**	N.R. **No**	**CIP4 Multiple phosphosites pSer296 in 15 s** **N-WASP: pSer426 1–2 min**
	Cholesterol, nystatin endocytosis [101]	Yes	N.R.	N.R.	
	EEA1, Rab5, GAPEX5, Rab31 [156–158]	Yes	N.R. **Yes**	N.R. **No**	**EEA1: pSer720 in 10 and 60 min;** **Rab5a: pSer193 — 60min** **GAPEX5: Multiple phosphosites pSer742, pSer1017 in 5–10 min Rab31: NPD**
	Ub, USP25* [159,160]	N.R.	N.R.	N.R.	**USP25: Transient pThr61 at 30 s**
	Caveolae* [116,161–163]	No	N.R. **Yes**	N.R. **No**	**Multiple phosphosites on Caveolin-1 pTyr phosphorylated within 30 s**
GLUT4 return to GSV compartment and sorting away from recycling endosomes	LRP1 [164]	Yes	N.R. **Yes**	N.R. **Yes**	**pSer4524 and pThr4525 at 60 min**
	IRAP [165–169]	Yes	Yes **Yes**	Yes **Yes**	**pSer51 in 5 min; pTyr70 high 1–10 min transient**
	Sortilin [170–173]	Yes	N.R. **No**	N.R. **No**	**pSer824 marginal (∼0.5) 15–20 min**
	GGA, AP1* [174–177]	N.R.	N.R. **Yes**	N.R. **No**	**GGA: pSer414/418 in 5 min LY only** **AP1(subunit mu): pThr152/154 in 30 s and 15 s resp. LY only** **Other AP1 subunit NPD**
	ESCRT [178]	N.R.	N.R. **No**	N.R. **No**	**Chmp3 and Vps4 NPD** **Chmp2b phosphorylated in 15 s**
	Retromer [166,179,180]	N.R.	N.R. **Yes**	N.R. **Yes**	**Vps35: pSer7 at 60 min** **Vps26a NPD** **Vps26b: multiple phosphosites and pSer302 in 2–5 min** **Vps29 NPD** **Snx3 and 27: some phosphorylation in 60 min. Not PI3K- or Akt-dependent**
	Golgin97 and Vti1a [170]	N.R.	N.R.	N.R.	**NISPD**
	Syntaxin 6* [181], Syntaxin 16 [182], mVps45 [183]	Yes (Stx16)	N.R. **No**†	N.R. **No**^†^	Stx16 dephosphorylation within 30min [181] **Stx16 some phosphorylation detected at long timepoints 60 min. Dephosphorylation for Ser35 detected since 15 s**
	Rab11, Rip11* [184,185]	Yes	Yes **Yes**	No **Yes**	**Rab11b: Multiple phosphosites some activated at 2–10 min** **Rip11: Multiple phosphosites, some pSer188 and 480 very fast 15 s**
	Rab4, KIF13 (motor protein) [135,186–188]	Yes	Yes **Yes**	N.R. **No**	Slow GTP loading over 45 min **Rab4 NPD** **Kif13: Fast 30 s multiple sites pThr1717**
	Rab14 [55,189]	N.R.	N.R. **No**	N.R. **Yes**	**2 min pSer97**
	TUG [190–192]	N.R.	N.R. **Yes**	N.R. **No**	Proteolytic cleavage in 10 min **pSer279 at 5 min**
	TRARG1(TUSC5) [193,194]	N.R.	N.R. **Yes**	N.R. **Yes**	**Multiple phosphosites Ser56, 70 and 72 in 20–60 min**
	TBC1D13, Rab35 [195]	N.R.	N.R. **No**	N.R. **Yes**	**TBC1D13: Ser184 in 20–60 min**
	*CHC22* [196]	N.R.	N.R.	N.R.	**NPD**
	Budding of GSV, *PLD* [61,62]	Yes	N.R.	N.R.	**PLD1 — NISPD**
Delivery of newly synthesised GLUT4 to GSVs and maintaining insulin responsiveness	Sortilin [170–172]	Yes	N.R.	N.R.	**See above**
	syntaxin 16, mVps45, syntaxin 6 [181–183]	Yes (Stx16)	N.R.	N.R.	Stx16 dephosphorylation within 30 min [181]. See above
	GGA, AP1 [174–177]	N.R.	N.R.	N.R.	See above
	ACAP1, Arf6 [197]	Yes	N.R.	N.R.	**NPD**
	VAMP7 (TI-VAMP), VAMP4 [198]	N.R.	N.R. **Yes**	N.R. **No**	**Vamp4 — Multiple phosphosites pSer17/20 in 1–5 min**
	Axin, TNKS [199–201]	N.R.	N.R.	N.R.	**Axin1 — multiple sites but below 0.5**
	P115 [202]	N.R.	N.R. **No**	N.R. **No**	**pSer717 slow 60 min**
	Sec16 [63,129,130]	N.R.	N.R.	N.R.	**See above**
	*CHC22* [196]	N.R.	N.R.	N.R.	NPD
	Transfected GLUT4 becoming insulin responsive	N.R.	N.R.	N.R.	6–9 h [174] 3.5 h in primary adipocytes [152]

Key molecules reported to modulate GLUT4 trafficking and their activation time scale and phosphorylation status in response to insulin stimulation and sensitivity to PI3K/Akt inhibitors (where known).

Italics text —proteins reported to have a role in skeletal muscle; N.R. *—* not reported.

Bold text *—* phosphorylation times and PI3K and Akt inhibitors data from Humphrey et al. [36]. Only values above the threshold of 0.5 log_2_ of the ratio Insulin-stimulated/unstimulated cells were reported.

NISPD *—* no insulin-sensitive phosphosites detected (protein listed in the database as a phosphorylated protein but no kinetic data available).

NPD *—* no phosphorylation detected (protein not listed in the database).

* Fast phosphorylation, less than 1 min, according to Humphrey et al. [36].

† As the inhibition of phosphorylation in [36] was assessed only at a time point of 20 min the effect of the inhibitors on transient phosphorylation at earlier timepoints or phosphorylation events occurring at later timepoints >20 min cannot be assessed.

In this perspective, we discuss how the different ‘phases’ of the GLUT4 trafficking responses to insulin and review kinetic considerations for how insulin signalling may co-ordinate multiple steps in GLUT4 traffic. We discuss methodological bottlenecks to a more complete understanding of the dynamics, molecules and systems involved in GLUT4 traffic, and suggest how we might accelerate identification of which trafficking steps, and regulatory proteins, are involved in signalling to the key phases of insulin action on GLUT4.

## The basics of insulin signalling to GLUT4

The proximal insulin signalling pathway required for GLUT4 translocation has been well characterised and very extensively reviewed elsewhere [7]. We, therefore, highlight only the basics of insulin signalling to GLUT4. Studies using the PI3K inhibitors wortmannin [8] and LY294002 [9] and an inhibitory and inactive PI3K p85 subunit [10,11] were among the first to show that PI3K activity is essential for insulin-stimulated glucose transport and GLUT4 translocation. Since then, studies using isoform-specific PI3K inhibitors have established that the main PI3K isoform involved is 1A [12,13]. The most important signalling event downstream of PI3K for GLUT4 translocation is the activation of the serine–threonine protein kinase Akt or protein kinase B (PKB) [14,15]. Akt is recruited to PIP3 at the PM through its PH domain with a similar time course to PIP3 generation [16], within ∼40 s of insulin binding to its receptor (Table 1), and this leads to Akt activation through the action of two kinases phosphoinositide-dependent kinase 1 (PDK1) [17,18] and mTOR complex 2 (mTORC2) [19]. Phosphorylation of Akt at both PDK1 and mTORC2 activating sites is required for full Akt activation [20,21]. Initial research on the role of Akt in insulin-stimulated glucose uptake and GLUT4 translocation relied extensively on genetic approaches including expression of constitutively active and membrane associated forms of Akt isoforms (Akt1, 2 or 3), siRNA knockdown in cell lines or knockout mouse models [22–26]. These demonstrated that Akt2 isoform expression and activity correlated with insulin-sensitive glucose uptake in insulin-sensitive tissues and is preferentially recruited to the PM in response to insulin stimulation [22,27,28]. More recently, specific inhibitors of Akt were developed (Akti-1/2 [29] and MK-2206 [30–32]), which confirmed that Akt activity is required for the very acute stimulation of glucose transport and GLUT4 translocation in response to insulin. Overall, these studies have provided strong evidence that the PI3K–Akt signalling pathway is very rapidly activated in response to insulin (Table 1), and is necessary and sufficient for insulin-stimulated GLUT4 translocation [24,33].

One of the fascinating features of insulin-regulated GLUT4 trafficking is that it requires the integration of signal transduction and subcellular protein traffic. But how does insulin signalling directly affect specific GLUT4 trafficking processes? Given the acute timeframe of insulin responses, and the key role for Akt in insulin-regulated glucose transport, the most likely mechanism is via protein phosphorylation. The discovery of the Rab-GTPase activating protein (GAP) TBC1D4 (AS160) as an Akt substrate established the first direct link between the insulin signalling and Rab-mediated control of membrane vesicle traffic [34,35]. Crucially, TBC1D4 phosphorylation is sensitive to PI3K and Akt inhibition, and occurs within 15 s of insulin stimulation, is maximal by 1 min (Table 1) [36]. Furthermore, a phosphorylation-dead mutant of TBC1D4 (termed TBC1D4-4P), has revealed that TBC1D4 phosphorylation by Akt is required for insulin-stimulated GLUT4 traffic [35]. These data, along with the kinetics of signalling to TBC1D4, are in keeping with a critical role for Akt-mediated TBC1D4 phosphorylation in acute insulin-stimulated GLUT4 translocation. TBC1D4 is a Rab-GAP, and negatively regulates GLUT4 translocation through inhibiting its substrates Rab10 and Rab14 in adipocytes (Rab8 and Rab13 are similarly implicated in muscle cells, Table 1). TBC1D4 maintains these Rabs in their inactive, GDP-bound, form, and inactivation of TBC1D4 following insulin signalling to TBC1D4 leads to GTP loading and Rab-mediated GLUT4 translocation.

## Considering kinetics of insulin-stimulated GLUT4 translocation

In early versions of the glucose transporter translocation model, it was assumed that the glucose transporters were delivered to the PM and then remained there until insulin dissociated from its receptor [2]. Using the hexose photolabel ATB-BMPA [8,37–40], some of the first studies on the kinetics of GLUT4 translocation revealed that GLUT4 continuously recycled from the PM to an endosome compartment and a sequestered intracellular-storage compartment even in the presence of insulin. These studies suggested at least 3-compartments are required to adequately account for GLUT4 traffic. They revealed that the main effect of insulin was to accelerate the rate of exocytosis from this sequestered compartment [37,40]. There also appeared to be small reduction in the endocytosis rate constant following insulin treatment. However, detailed follow-up studies revealed that, when considering GLUT4 endosomal recycling, there was no insulin-dependent reduction in GLUT4 endocytosis [41].

In addition to insulin primarily targeting GLUT4 exocytosis, studies on photolabel-tagged GLUT4 revealed that there are (at least) two phases to the insulin response. First, there is a *fast acute* phase of the response to insulin where, within ≈3 min, 50% of GLUT4 is delivered in a burst to the cell surface [37,42,43], likely from its specialised storage compartment termed GLUT4 storage vesicles (GSVs). Second, there is *steady-state* phase, which can last for hours, where elevated PM GLUT4 abundance is maintained via GLUT4 recycling to the PM with a half time of ≈8 min [37,42,43].

The concept of two phases of the GLUT4 trafficking response to insulin, and of insulin signalling increasing GLUT4 exocytosis rates, are consistent with experimental data from total internal reflectance fluorescence microscopy (TIRF-M). TIRF-M has allowed study of the kinetics of GLUT4 movement close to the PM [44–48], and the application and use of pH-sensitive GLUT4 constructs (e.g. phluorin-GLUT4) [45,49] has enabled study of GSV fusion at this site. Studies in adipocytes have revealed a burst of GLUT4 vesicle fusion events in the first 5–10 min directly after insulin addition. This burst of fusion activity then falls away to a steady-state rate of vesicle fusion that remains higher than observed in unstimulated cells [45,49].

The presence of two phases to the insulin response was also supported by studies led by James and Coster using antibody binding to epitope-tagged GLUT4 reaching the cell surface of 3T3-L1 cells to monitor GLUT4 traffic [50–52]. These studies revealed that GLUT4 appeared to be released to the cell surface in a graded manner to increasing doses of insulin, with a distinct plateauing and saturation of antibody binding in the basal state and at submaximal insulin levels. In the basal state only ≈20% of GLUT4 recycled and bound cell surface antibody while 80% appeared to remain in a non-recycling compartment. Submaximal levels of insulin lead to a quantal increase in the antibody saturation point, but a maximal insulin stimulation was necessary to mobilise all the available GLUT4. The concept of a static-retention GSV compartment is interesting since it suggests that insulin acts to release GLUT4 from a highly sequestered and non-recycling pool, effectively increasing the mobility of an additional proportion of sequestered GLUT4 available to traffic to the PM.

**Figure 1.**
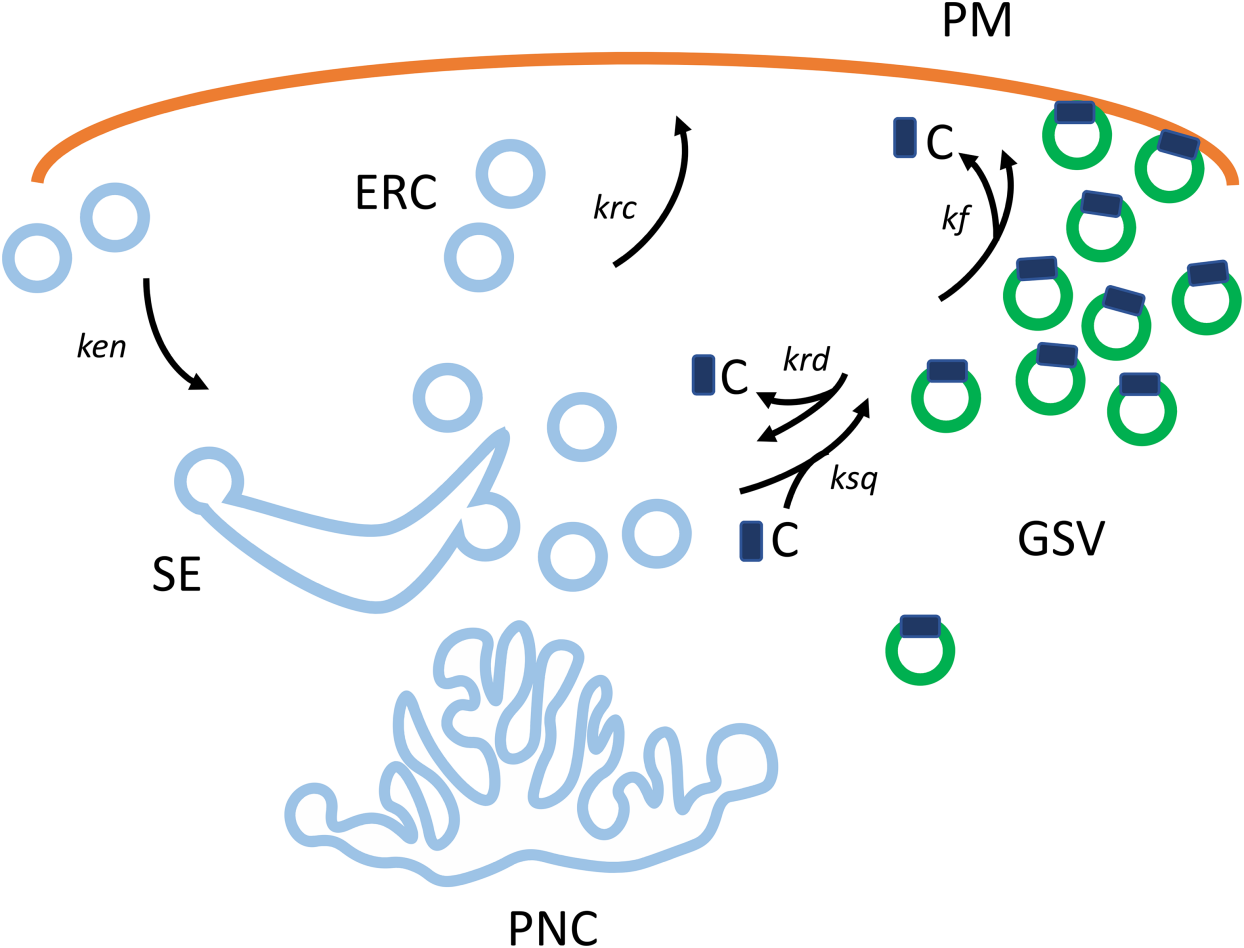
GLUT4 sequestration and exocytosis. The schematic diagram represents an extension of the 3-compartment sequestration model [42] and the 4-compartment dynamic-retention model [54,55]). The compartments considered in modelling of kinetics of GLUT4 traffic are the plasma membrane (PM), a sequestered GLUT4 vesicle (GSV) compartment, sorting endosomes (SE) and perinuclear compartments (PNC) and an endosome recycling compartment (ERC). The GSV reservoir is supplied by vesicles from SE and PNC, but the proportion of vesicles supplied by these compartments will vary in different cell types. In basal 3T3-L1 cells ∼80% of the GLUT4 is present in GSV, while 20% of GLUT4 is recycled from SE through ERC to the PM [54]. We suggest here that a retention-catalyst C is responsible for the partitioning and saturation of the sequestered GLUT4 compartment. It is proposed that C catalyses formation of GSV. In addition, C is released from the GSV compartment when insulin signalling leads to vesicle fusion with the PM. The rate constants linking these compartments are: *kseq* for movements at the saturable step and requiring C; *kf* for fusion of docked vesicles with the plasma membrane — and associated with desaturation of the GSV compartment and release or recycling of C; *krd* for reversal of docking without fusion — this step involves vesicles that sample docking sites but do not fuse; *krc* for recycling of GLUT4 from SE through ERC and to the PM.

Building on these data on the quasi-static GSV compartment, Mastick and co-workers [41,53], together with Coster and co-workers [54–56], developed a 4-compartment dynamic-retention kinetic model for GLUT4 traffic. The main feature of this model is that of two exocytotic routes to the PM, one from a sequestered GLUT4 compartment and one from an endosome recycling compartment (ERC) (Figure 1). In the absence of insulin, it is proposed that GLUT4 exocytosis mainly occurs through the recycling endosome route (≈20% of GLUT4), with the bulk of the GLUT4 (≈80%) sequestered and partitioned from this route into the more slowly recycling GSV compartment. This 4-compartment model can be successfully used to simulate quantal effects in release of GLUT4 and plateauing of cell surface antibody binding to GLUT4. Insulin signalling is proposed to stimulate both the release of GLUT4 from its sequestered compartment (a TBC1D4/Rab10 regulated step) and fusion of GSVs with the PM [54–56]. These modelling studies also accounted for insulin responsive traffic of transferrin receptors from sorting endosomes and LRP1 traffic through an ERC [41]. Both endosome ligand recycling compartments were kinetically separate from the sequestered GLUT4 storage compartment.

It is noted in Supplementary S1a that the simpler 3-compartment sequestration model can also be used to simulate plateauing of antibody binding responses. This occurs when the model includes a endosome recycling step (as discussed in [42]) and therefore two exocytosis steps (one from sequestered GSV and one from endosomes). However, none of the three models above has given a mechanistic basis for the level of saturation of antibody binding and for partitioning of compartments in the basal state. We suggest that the concentration of sequestered GLUT4 is dependent on the level of a retention-catalyst (such as a vesicle coat protein or a retaining Rab protein), and include this suggestion as an extension to the 3-compartment model [42]. This additional feature is shown schematically in Figure 1, and further developed in Supplementary 1b. In the basal state, the availability of the retention-catalyst limits the concentration of GLUT4 in the non-recycling (or slowly recycling) compartment (at ≈80% in 3T3-L1 cells). In this basal state the level of free catalyst approaches zero, as all the available catalyst is bound to the GSV. It is proposed that insulin signalling leads to a release of GLUT4 vesicles from the retention-catalyst for fusion with the PM in a rapid burst. This leads to increased delivery of GLUT4 at the PM, but also releases the retention-catalyst to regenerate the GSV compartment. This updated 3-compartment model provides an approach to directly link insulin signalling molecules and catalysis to compartmentalisation and movement of GLUT4 vesicles.

## The site of action for PI3K–Akt–TBC1D4 signalling in GLUT4 trafficking

In addition to the observations of the phases of the insulin response, TIRF-M studies have provided detailed analysis of GSVs behaviour at the cell surface (e.g. trafficking to the cell periphery, docking, fusion) and in the PM bilayer (e.g. dispersal). These studies have revealed that insulin acts to increase vesicle docking and fusion at the PM. Movement of GLUT4-containing vesicles into the TIRF zone was found to be wortmannin-insensitive. However, wortmannin markedly reduced GLUT4 vesicle docking, demonstrating that the capture of GSVs by the PM is dependent on PIP3 signalling [44]. A cell-free fusion assay, which combines separate PM, intracellular vesicle and cytoplasm fractions from basal or insulin-stimulated cells, also revealed that insulin facilitates fusion of GSVs with the PM. In this assay, wortmannin was inhibitory in the overall cell-free fusion system [57,58]. This assay does not distinguish whether the docking or fusion step is involved in the inhibitory response to PI3K-inhbition, but these data are consistent with the docking/fusion of GLUT4 vesicles at the PM as a key regulated process downstream of PIP3 synthesis.

There is also evidence that TBC1D4 may play a role in the docking/fusion of GSVs at the PM. First, the phospho-tyrosine binding domains of TBC1D4 inhibited GLUT4 vesicle fusion with the PM in a cell-free assay [58]. Second, TIRF-M studies revealed that TBC1D4-4P markedly reduced GLUT4 vesicle docking and consequently fusion [59,60]

Away from actions at the PM several studies have shown insulin effects on vesicle budding from the perinuclear compartment [61,62]. Brumfield *et al*. [63] provided evidence that insulin/Akt signalling promoted GLUT4 mobilisation from the perinuclear region. Using a photoconvertible GLUT4 reporter, they measured a ∼1.5-fold increase in the presence of insulin in GLUT4 movement from this region over a time course (20 min) consistent with a role in delivering GLUT4 to the PM. Because photoconversion can be confined to specific subcellular locations this technique can, to some extent, be considered as a providing a separation and isolation from other ongoing processes in other locations. In addition, the Akt inhibitor MK2206 inhibited this insulin response. Knockdown of TBC1D4 increased GLUT4 mobilisation from the perinuclear region to the PM [63]. In contrast, knockdown of the TBC1D4 cognate Rab, Rab10, had the opposite effect. This suggests that insulin signalling to TBC1D4/Rab10 promotes GLUT4 delivery to the PM to sustain the insulin response. Together, these data support a role for GLUT4 vesicle delivery to the cell periphery as an insulin-regulated step. However, the extent of insulin activation (fold-change) of this perinuclear step is smaller than those at the PM.

The experimental data outlined above suggest that PI3K–Akt signalling acts at multiple sites in GLUT4 trafficking, including traffic from the perinuclear region and GLUT4 vesicle docking and fusion at the PM. In addition, modelling and direct experimental evidence have suggested a role for TBC1D4 in release of GLUT4 towards the cell periphery [63], the sorting of GLUT4 into GSVs [56], release of GSVs to the PM [59] together with GSV interactions with the PM [44,57–60]. Does insulin signalling act via PI3K–Akt–(TBC1D4) to target several different processes in GLUT4 traffic?

In section ‘Considering kinetics of insulin-stimulated GLUT4 translocation’, we have proposed an updated model of GLUT4 traffic that includes a ‘retention-catalyst’ that is required for GSV formation and sequestration. Importantly, this catalyst is limiting, so that the size of the GSV pool of GLUT4 is finite. This ‘retention-catalyst’ could be the TBC1D4–Rab10 couple. If this is the case, then PI3K–Akt catalysed suppression of TBC1D4 Rab-GAP activity and increased Rab-GTP loading will have two immediate consequences: (1) release of GSVs for fusion with the PM and (2) desaturation of the GSV compartment and refilling of this compartment. As such, this proposed catalytic cycle involving a retention-catalyst (Figure 1) may offer advantages in terms of fully understanding the apparent multiple sites of action of insulin action occurring through TBC1D4.

However, control of GLUT4 by TBC1D4 alone provides an incomplete picture of insulin action on GLUT4 traffic. Some studies indicate that vesicle fusion can be perturbed or activated independently of TBC1D4-driven vesicle docking [28,60,64]. Indeed, many molecules that are involved in SNARE complex formation and actin remodelling could be involved in activation of fusion independent of the involvement of TBC1D4 in vesicle docking. These proteins are targets of insulin-stimulated phospho-serine/threonine and of phospho-tyrosine kinase activities (Table 1 and section ‘Identifying insulin control of GLUT4 traffic via phosphorylation’).

In addition to these findings related to GLUT4 vesicle docking and fusion, detailed analysis of GLUT4 movement within the PM using TIRF-M suggests that insulin regulates GLUT4 dispersal from fusion sites [47,49,65,66]. The significance of this process for GLUT4 catalytic activity needs further resolution and new methods to study these steps independently of other events at the PM are needed. To some extent, an altered dispersal within the PM may be a prelude to GLUT4 interaction with clathrin lattices and internalisation [49].

## Identifying insulin control of GLUT4 traffic via phosphorylation

A major goal of the field remains to understand how insulin signalling controls GLUT4 traffic at the molecular level. TBC1D4 is the most highly studied substrate of insulin signalling in this context, and the data generated on TBC1D4 highlights the power of combining effective experimental tools to manipulate insulin signalling (e.g. TBC1D4-4P mutant) with experimental systems that discern specific GLUT4 trafficking processes such as vesicle docking.

As described above, insulin regulates GSV interactions and fusion with the PM. Consistent with this, some proteins implicated in GSV docking/fusion are direct Akt substrates or regulated downstream of Akt substrates. These include Rab10 [67], myosin Va [68], synip [69], tomosyn [70] and RalA [71]. In contrast, the activities of other mediators of GLUT4 vesicle-PM interactions (such as Munc18c [72], Rab3 [73], TC10 [74] and exocyst components [75–77]) are regulated by insulin, but not affected by PI3K or Akt inhibitors (Table 1). In addition to insulin regulation of serine/threonine kinases, tyrosine kinases, including the insulin receptor are also implicated in the important docking and fusion steps of insulin action. The formation of the ternary SNARE complex at the PM is reported to be targeted by insulin signalling via Munc18c. The GSV docking and the fusion steps involve the formation of the SNARE complex between the t-SNAREs syntaxin4 and SNAP23 [78–81] at the PM and v-SNARE protein VAMP2 on GSVs. The formation of this complex is stimulated by insulin [82], which fits with the stimulatory effect of insulin on GSV docking and fusion reported in the TIRF studies and indicated as being critical sites in kinetic modelling studies. This process is modulated by Munc18c [82–87], which is now known to be a direct target of the tyrosine kinase activity of the insulin receptor [88]. Furthermore, an interesting and powerful new approach to studying and isolating insulin action on SNARE proteins has been developed using proximity ligation assays. This approach has been used to show that insulin-stimulated Munc18c phosphorylation is required for SNARE assembly [89]. Phosphomimetic mutants (Y_219_E or Y_521_E), but not phosphodeficient mutants (Y_219_F or Y_521_F), rescued defective insulin-regulated GLUT4 traffic in Munc18c knockdown adipocytes, suggesting that phosphorylation of this protein promotes SNARE complex formation [72,90,91]. In addition to Munc18c, Syntaxin 4 is phosphorylated in response to insulin, and both Syntaxin 4 and Munc18c are phosphorylated rapidly, with time courses of seconds and minutes (detailed in Table 1). These data are consistent with a role for signalling to these proteins in the burst of GSV exocytosis upon insulin stimulation.

Phospho-proteomics data provide unbiased insight into new sites of insulin action. Global analysis of the kinetics and PI3K/Akt dependence of the insulin signalling network in 3T3-L1 adipocytes revealed very rapid activation of the PI3K–Akt pathway (within 1 min) and that PI3K activation is required for a large proportion (∼66%) of all insulin-stimulated protein phosphorylation/dephosphorylation events in these cells [36]. Furthermore, this study uncovered the remarkable breadth of the insulin signalling network with approximately 5000 phosphosites on 2000 proteins that are regulated by insulin. This list contains phosphosites on known regulators of GLUT4 traffic that are not functionally characterised (Table 1), including some on Rabs and their regulatory or effector proteins. Rabs are G-proteins and their activity can be controlled by insulin through combinations of phosphorylation of GEF and GAP proteins that modulate GTP loading and by direct phosphorylation of the Rab themselves. Many of these phosphorylation reactions occur rapidly (Table 1). We know that the Rab10 GAP (TBC1D4) and GEF (DENND4C) are targeted by insulin signalling [35,92], but phospho-proteomics has revealed that Rab10 itself also undergoes insulin-regulated phosphorylation [36] (Table 1). It will be of interest to ascertain how Rab10 phosphorylation influences Rab10 GTP loading and activity. Some data suggest that Rab10 on GLUT4 vesicles may not be GTP-loaded in response to insulin [67]. However, it is technically challenging to determine Rab protein GTP loading status and we require more refined techniques for determining both levels of the GTP loading and the activation of subpopulations of Rab proteins within cells. This is also relevant to additional Rabs since many Rabs and Rab effectors are phosphorylated rapidly by insulin (Table 1) including Rab11/Rip11, Rab3/Rab3Gap1/Rab3ip and RAB13/MICAL-L2. Rab5/RABEX5 is also subject to insulin-stimulated phosphorylation, albeit with slower kinetics (Table 1).

Overall, there are many very rapidly phosphorylated insulin-regulated proteins that are known regulators of GLUT4 traffic (Table 1). However, we note that phospho-proteome screening has revealed phosphosites on proteins that are implicated in GLUT4 vesicle traffic but have not yet been extensively studied as targets of insulin action. Many of these under-studied proteins are rapidly phosphorylated by insulin signalling often in <1 min (see asterisk in Table 1). It is, therefore, clear that phospho-proteomic analyses are an invaluable resource, but also present the challenge of firstly filtering out sites of greatest interest, and then understanding the functional significance of these phosphorylation events in GLUT4 translocation.

## Further directions (or where to next?)

Method development to allow distinct GLUT4 trafficking processes to be studied in isolation and in sufficient kinetic detail are critical for us to fully map the major sites of insulin action in GLUT4 trafficking. We propose that by focusing on insulin-regulated trafficking steps, and identifying putative regulatory signalling events from new or existing phospho-proteomics data, we will accelerate experimental elucidation of the mechanism of insulin action.

One avenue for method development we are particularly interested in is cell-free experimental systems. Although difficult to set up, they have yielded big advances in the study of isolated SNARE involvement in neuronal fusion systems [93–95]. This has now been further expanded and the SNARES involved in GSV fusion have been successfully used for characterising proteins involved in the docking and fusion events [94,96,97]. With the advent of a range of more sensitive fluorescent molecules and fluorescent nanodots, perhaps new and improved cell-free assays of insulin action on fusion can be developed to provide the mechanistic isolation of individual reactions that are not possible in the whole cell setting. The PM lawn assay and permeabilised cell assays [98] also have potential as cell-free approaches to studying vesicle docking and fusion. Lawns which retain activity after attachment to coverslips may be accessible to live TIRF microscopy. Isolated adipocyte attached to coverslips form regular arrays of isolated PM sheets because of the regular packing of large cells. These cells can readily be lysed to remove the bulk of cell content [43]. GLUT4 vesicle reactions with isolated lawns could be followed under conditions in which the cytosol components of the fusion reaction are modified.

One caveat to use phospho-proteomics data is that there are currently very few studies on separately manipulating specific phosphorylation sites on target proteins or of identifying and separating functional from non-functional phosphorylation [99]. These studies will be vital, especially for proteins with multiple insulin-regulated phosphorylation sites. A nice example of studying isolated processes is a recent series of experiments in which recombinant full length TBC1D4 was incubated with isolated and purified specific kinases [100]. This study showed the preference of specific phosphorylation sites for the upstream kinases Akt and AMPK, but also that a major effect of TBC1D4 phosphorylation induced by these kinases was a reduction in TBC1D4 binding to IRAP. This study supports the model whereby insulin signalling disrupts TBC1D4 binding to GSVs (via IRAP). Overall, phospho-proteomics datasets are a valuable resource to find new regulatory mechanisms underpinning insulin-stimulated GLUT4 traffic, but it will require substantial effort to assess the role of high priority phosphorylation sites in insulin signalling to GLUT4. Related to this, we should not lose sight of the usefulness of small molecule inhibitors as an important part of the data presented in this review came from their use. Although it is important to be mindful of off-target effects [31], small molecules generally act rapidly and can be used on timescales similar to that at which insulin acts to provide clear data on the involvement of PI3K or Akt, for example, in the insulin response.

We suggest that considering retention-catalysts may help begin to unpick how insulin signalling regulates steps of GLUT4 traffic. Here, we propose that saturation of the GSV compartment occurs because of a limit in retention-catalyst availability. Exploration of the consequences of saturation of membrane traffic compartments and steps could be more extensively studied in future and could apply to several (or even all) steps in complex pathways. Their consideration may simplify the considerations of membrane compartment partitioning (and the associated rate constants for traffic through these compartments), as compartment size may be restricted by separate facilitators at each step. For example, clathrin lattices [98] and clathrin vesicle coating could conceivably be limited at the PM and lead to partitioning between a clathrin and non-clathrin routes for endocytosis [101]. This concept also has implications in experimental situations systems in which a recombinant mediator is introduced. The endogenous saturation point for a compartment may be exceeded and there may be overflow into compartments that would not normally be used in an unperturbed system. For example, in studies in which GLUT4 has been overexpressed [102], it exceeds the capacity of the insulin-regulated storage system and more GLUT4 is diverted to a recycling system that is not insulin regulated. The GLUT4 is diverted to the PM and raises basal levels of unregulated traffic.

From kinetic data available to-date, it is clear that GSV docking and fusion are major sites of insulin regulation in adipocytes. Furthermore, evidence from human adipose tissue suggests that the tethering/docking and fusion steps are impaired in insulin resistant adipocytes [103]. It is important to note that these kinetic considerations also apply for insulin action in muscle cells (the major site of insulin-stimulated glucose uptake). GLUT4 vesicles were found to be tethered (or docked) at the sarcolemma and transverse tubule membranes in the absence of insulin, and fused with the limiting membrane from these sites in response to insulin [46,104,105]. These data together suggest that it is critical to focus attention on molecules and docking/fusion processes at the PM in both fat and muscle. This will help to map out how insulin signalling impinges upon delivery of GLUT4 to its functionally and physiologically important location and delivers enhanced glucose transport into insulin target cells.

## Open Access

Open access for this article was enabled by the participation of University of Bath in an all-inclusive *Read & Publish* pilot with Portland Press and the Biochemical Society under a transformative agreement with JISC.

## Abbreviations

ERC: endosome recycling compartment
GSVs: GLUT4 storage vesicles
mTORC2: mTOR complex 2
PDK1: phosphoinositide-dependent kinase 1
PM: plasma membrane
TIRF-M: total internal reflectance fluorescence microscopy
