# Supplementary material for: GLUT4 On the move
Source: Biochem J. 2022 Feb 11;479(3):445–62. doi: 10.1042/BCJ20210073 (PMC8883492; doi:10.1042/BCJ20210073)
Supplement: Supplementary Material [file BCJ-479-445-s1.pdf]

Supplement S1a

Quantal and plateauing/saturation effects in antibody binding experiments

The importance of a partitioning of GLUT4 vesicles into those which were recycling and those which were non-recycling in the basal state was highlighted in antibody binding experiments. A large non-recycling basal **GSV** compartment (saturating and partitioning at ≈80% of GLUT4) in 3T3-L1 cells was described [1]. These data were later analysed using a 4-compartment dynamic-retention model in which the sequestered compartment **GSV** was described. Rate constants included in this model described slow influx and efflux to and from this large **GSV** compartment and, in addition, there was faster recycling back to the plasma membrane from sorting endosomes and through an endosome recycling compartment (**ERC**) [2, 3]. Collectively, these studies showed that these rate constants would simulate the plateauing or saturation of antibody binding to GLUT4 under basal and submaximal insulin concentrations.

We show here that the simpler 3-compartment sequestration model [4] can also simulate the saturation of antibody binding to GLUT4. This simpler model (with just 3 compartments and no separate ERC compartment as in the dynamic-retention model) has two exocytosis routes, one through the sequestered GLUT4 storage pool and the other through endosome recycling (called *rken*, or *rkc*) and a single rate constant describing the complete flux through **ERC** to the **PM**. To demonstrate the ability of this model to account for antibody binding data, we have first simulated the antibody binding that would occur in basal 3T3-L1 cells using the rate constants described for the reference 4-compartment model [2, 3]. Then, we curve fitted the simpler, 3-compartment sequestration model to these data (Fig. S1a). An excellent match is obtained. In addition, we have used curve fitting to demonstrate that the model can be used to analyse basal antibody binding data in L6 muscle cells [5].

Figure S1a
The 3-compartment sequestration model can account for a plateauing/saturating effect in antibody binding in 3T3-L1 adipocytes and in the muscle cell line L6.

The rate constants used in these simulations are those listed in the scripts below (S1c). The simulated 3T3-L1 curve from the sequestration model (green line) closely match the reference data curve from the dynamic-retention model [3](broken red-line line). The simulated curve (blue line) was obtained from a fit to antibody-binding data in basal L6 cells [5].The curves are plotted as a fraction of maximal binding that occurs either with a maximal insulin concentration in 3T3-L1 cells or a combined effect of insulin and AICAR (which stimulates AMP-activated kinase) in L6 cells.

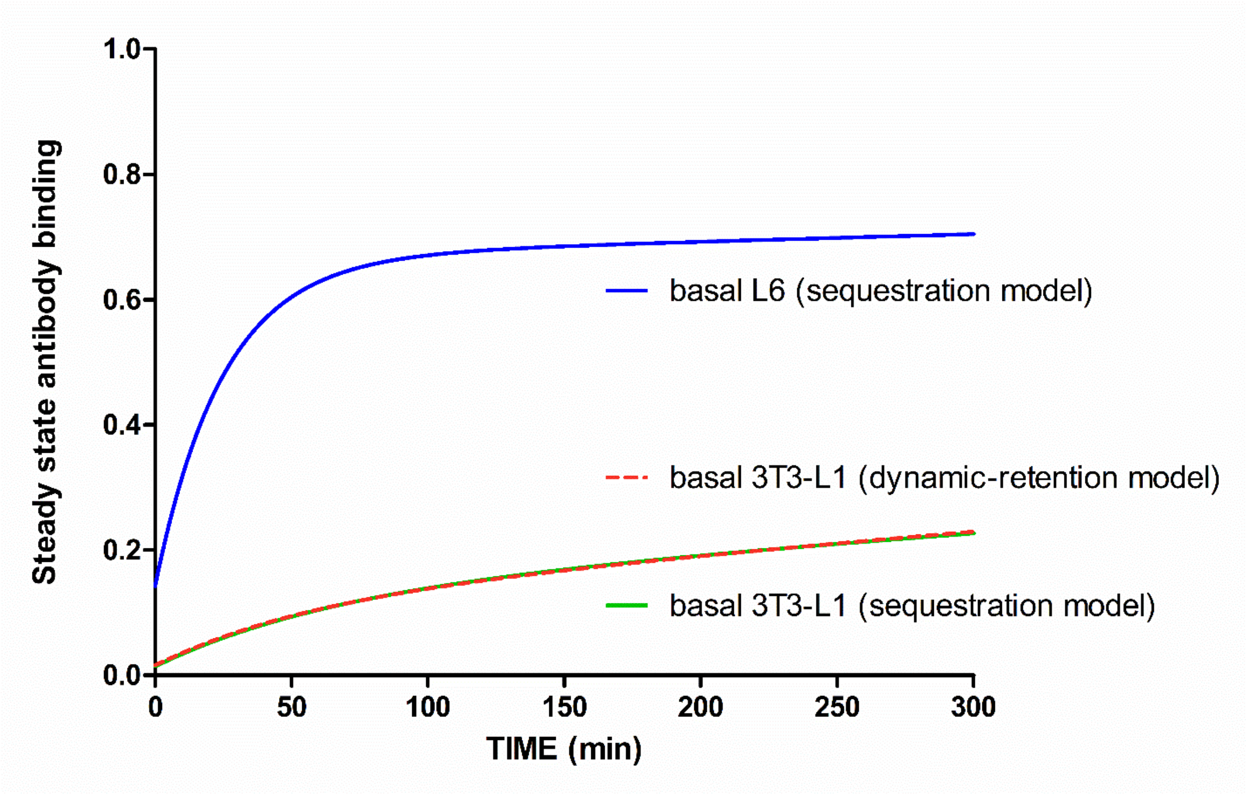

Supplement S1b

A catalysis model for insulin stimulated GLUT4 subcellular traffic

To analyse the effects of the effects of a catalyst on GLUT4 sequestration we assume just 3 major compartments (**Fig. 1**, main article text): the plasma membrane (**PM)**, endosomes or sorting endosomes (**SE**) and a sequestered GLUT4 compartment (**GSV)**. These are essentially the same compartments as previously described in the 3-compartment sequestration model [4] but here we extend this model by inclusion of a catalyst. To simplify the mathematical treatment, we assume **SE** includes vesicles derived from a perinuclear compartment. The common feature is the interaction with the catalyst that may occur in different regions of the cell. The proportions of GLUT4 in different pre-fusion compartments may differ between cell types.

In this model, movements of vesicles from **SE** to **GSV** are catalysed by a process involving **C** (a catalyst). **C** could be, for example, a key coat protein or a Rab protein together with an enzyme such as a Rab-GAP. This kinetic treatment therefore provides a link between movement of GLUT4 vesicles and insulin signalling. **C** is considered to reform as a consequence of fusion of **GSV** with **PM** (or possibly some other process that leads to fusion such as opening of a docking pore, interaction of the docked vesicles with motor proteins or microfilaments etc). As **C** increases following fusion and desaturation of **GSV**, the replenishment of **GSV** is facilitated by the recycling of **C**.

Catalysis by **C** is limited by Co (the sum of the concentration of free **C** and **C** that is attached to **SE** vesicles). Co is a partitioning factor and is approximately equal to the maximum level at which **GSV** saturation occurs in the basal state (≈80% in **GSV**). The rate constants that link GLUT4 compartments are: *ksq* - a saturable step requiring **C** as catalyst and leading to sequestration of GLUT4 vesicles that are primed for docking (this second-order rate constant is a replacement for the first-order rate constant *ksq* in previous modelling [4]); *kf* - fusion of **GSV** with the plasma membrane releasing the catalyst or binding sites (this step is a replacement for *kex* in previous modelling); *krd* - reversal of docking or vesicle sampling the docking site without fusion and then returning to **SE** with release of **C**; *krc* – recycling from sorting endosomes through the endosome recycling compartment (labelled as **ERC** Fig. 1 main text) to the **PM**.

The equations describing movements of GLUT4 through these compartments are:

$$\mathrm{Co} = \mathbf{C} + \mathbf{GSV}$$

$$\frac{d}{dt}(p) = \mathbf{SE}.krc + \mathbf{GSV}.kf - \boldsymbol{P}.ken$$

$$\frac{d}{dt}(se) = \mathbf{P}.ken + \mathbf{GSV}.krd. - \mathbf{SE}.(krc + ksq.\mathbf{C})$$

$$\frac{d}{dt}(gsv) = \mathbf{SE}.ksq.\mathbf{C} - \mathbf{GSV}.(kf + krd)$$

These equations are numerically integrated to generate simulations of transitions from one steady state to another (usually from the basal state to the insulin-stimulated state). Algebraic solutions for the 4 simultaneous equations with d/dt = 0.0 give the initial fractional concentrations. The transitions involve an exponential term plus a desaturation (or saturation)

term [6]. However, numerical integration solutions can also be used for curve fitting to transition time course data. Of most significance, the steady state equation describing the concentration of **GSV** is:

$$\mathbf{GSV} = \mathrm{Co}.\frac{\mathbf{SE}}{\frac{kf+krd}{ksq}+\mathbf{SE}} \qquad \text{Eqn.1}$$

Eqn. 1 describes the binding reaction between **SE** and **C** and the level of saturation of **GSV**. In the basal state the fractional saturation is close to 1 (fully saturated). Therefore **GSV** ≈ Co (the partition factor) and **C** is close to zero.

Both the rate of formation of **GSV** from **SE** and the rate of release of the **GSV** to **the PM** (v(in) and v(out), respectively) are given by a single equation:

$$\mathrm{v(in)} = \mathrm{v(out)} = kf.\mathrm{Co}.\ \frac{\mathbf{SE}}{\frac{kf+krd}{ksq}+\mathbf{SE}} \qquad \text{Eqn.2}$$

Eqn. 2 is a Michaelis Menten equation for a saturable enzyme (or binding site) with a maximum rate equal to Co.*kf* and a dissociation or affinity constant equal to (*kf* + *krd*)/*ksq).* In the basal state the fusion of **GSV** with the **PM** will be very low (*kf* is very low in the basal state). The rate of formation of **GSV** is also very low as the availability of **C** is very low in the basal state. Therefore, the **GSV** compartment is quasi-static with a high concentration of GLUT4 vesicles but only a slow flux through the compartment. A rapid increase in *kf* due to insulin action would increase release from **GSV** to **PM** but would also lead to desaturation and an increase in **C** (which allows the **GSV** to be replenished with vesicles from **SE** using recycled **C**). The low affinity constants for the simulation in **Fig S1b** are consistent with high stationary-vesicle/mobile-vesicle ratios observed the TIRF-M study by Lizunov *et al.* [7].

Figure S1b. Catalyst model for the transition from basal to insulin-stimulated steady states in 3T3-L1 cells.

The **GSV** compartment (orange line) in the basal state in 3T3-L1 is initially close to saturation (at ≈85% of total GLUT4) and the level of the catalyst **C** (red line) not already bound to GSVs is therefore very low. Following an insulin-stimulated increase in fusion of vesicles, desaturation of **GSV** occurs delivering GLUT4 to the plasma membrane (**PM**, green line). **PM** increases to a new steady state level (at ≈20% of total GLUT4). This process replenishes **C** and therefore allows vesicles from the sorting endosome (**SE**) compartment (dashed blue line) to bind **C** and catalyse refilling **GSV**. A reference curve was generated for the transition from the basal to the insulin state in 3T3-L1(the dynamic-retention model) using parameter values from Brewer et al [3] (dark green dashed line). In the catalysis model, the *kf* rate constant alone controls both emptying of **GSV** (by fusion of vesicles with **PM)** and the refilling of **GSV**, as both processes are dependent on the availability of **C**. Parameters for the reference dynamic-retention model and the Catalysis model can be compared in Supplement Section 1c - Script 2 and Script 3, respectively.

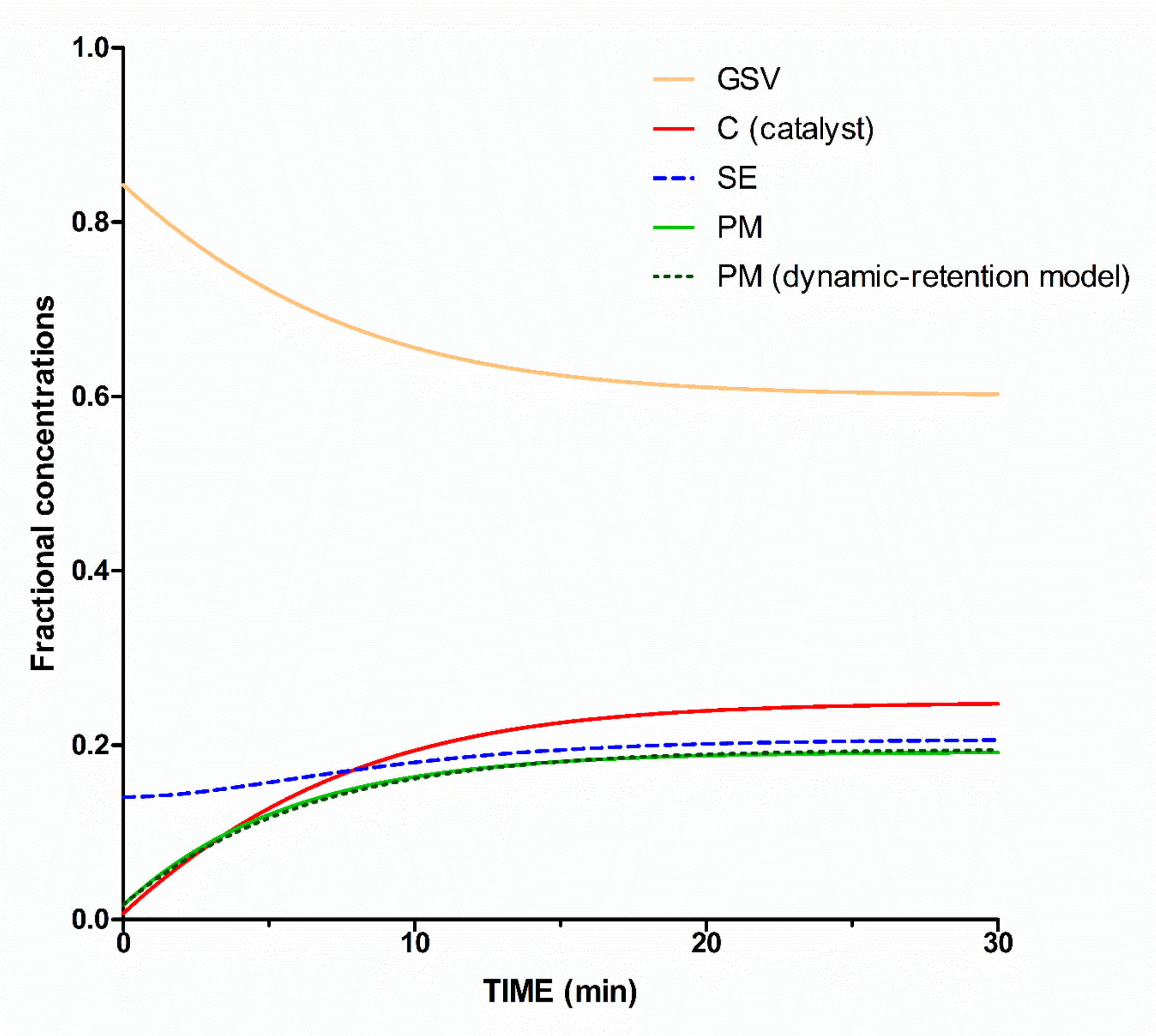

Supplement S1c - Scripts used in model simulations

The scripts below were generated for use with Berkley Madonna v8. The simulation of the dynamic-retention model was also compiled for Matlab (not shown) to check for accuracy using alternative software. Comments are between { } brackets

1. Script for simulation and integration of rate equations – 3-compartment sequestration model [4]

{

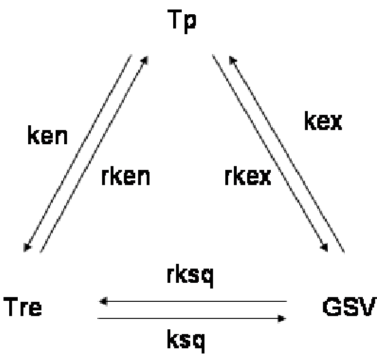

Tp, Tre and GSV are the plasma membrane, the recycling endosomes and the sequestered GLUT4 Storage Vesicle (GSV) compartments, respectively is the plasma membrane}

{numerical integration of simultaneous differential equations}
d/dt (tp) = rken*tre+kex*gsv-tp*(ken+rkex)
d/dt (tre) = tp*ken+rksq*gsv-tre*(rken+ksq)
d/dt (gsv) = tp*rkex+tre*ksq-gsv*(kex+rksq)

d/dt (lp) = rken*ulre + kex*ulgsv
d/dt (ulre) = rksq*ulgsv - ulre*(rken+ksq)
d/dt (ulgsv) = ksq*ulre - ulgsv*(kex+rksq)

{calculation of initial fractional concentrations of intermediates using King-Altman method}
tot=kex1*(rken1+ksq1)+rksq1*rken1+ken1*(kex1+rksq1)+rkex1*rksq1+ksq1*(ken1+rkex1)+rken1*rkex1
INIT tp=(kex1*(rken1+ksq1)+rksq1*rken1)/tot
INIT tre=(ken1*(kex1+rksq1)+rkex1*rksq1)/tot
INIT gsv= (ksq1*(ken1+rkex1)+rken1*rkex1)/tot

INIT lp =(kex1*(rken1+ksq1)+rksq1*rken1)/tot
INIT ulre =(ken1*(kex1+rksq1)+rkex1*rksq1)/tot
INIT ulgsv = (ksq1*(ken1+rkex1)+rken1*rkex1)/tot

{State 2 parameters -steady state}
ken = ken1
kex = kex1
ksq = ksq1
rken = rken1

rkex = 0.0
rksq = 0.0

{State 1 parameters - L6 basal}
ken1 = 0.15
kex1 = 0.0004
ksq1 = 0.00025
rken1 = 0.0402
rkex1 = 0.0
rksq1 = 0.0

{State 1 parameters – 3T3-L1 basal}
ken1 = 0.15
kex1 = 0.0004
ksq1 = 0.00247
rken1 = 0.01306
rkex1 = 0.0
rksq1 = 0.0

2. Script for simulation and integration of rate equations – 4-compartment dynamic-retention model [2, 3]

{P, GV, SE and RE are the plasma membrane, GLUT4 vesicle, sorting endosome and recycling endosome compartments, respectively}

{Integration equations}
d/dt (P) = kfg*GV + kfe*RE - ken*P
d/dt (GV)= ksq*SE - kfg*GV
d/dt (SE) = ken*P - (ksq + kso)*SE
d/dt (RE) = kso*SE - kfe*RE

d/dt (LP) = kfe*ULRE + kfg*ULGV
d/dt (ULGV)= ksq*ULSE - kfg*ULGV
d/dt (ULSE) = -ksq*ULSE - kso*ULSE
d/dt (ULRE) = kso*ULSE - kfe*ULRE

{Initial fractional concentration values}
tot = kso1*kfe1*kfg1+ksq1*kfg1*kfe+ken1*kfg1*kfe1+ken1*ksq1*kfe1+ken1*kso1*kfg1
init P = (kso1*kfe1*kfg1 + ksq1*kfe1*kfg1)/tot
init SE = ken1*kfg1*kfe1/tot
init GV = ken1*ksq1*kfe1/tot
init RE = ken1*kso1*kfg1/tot

init LP = (kso1*kfe1*kfg1 + ksq1*kfg1*kfe1)/tot
init ULSE = ken1*kfg1*kfe1/tot
init ULGV = ken1*ksq1*kfe1/tot
init ULRE = ken1*kso1*kfg1/tot

{State 2 – basal steady state}
ken =ken1

```
kfe = kfe1
kfg = kfg1
kso = kso1
ksq = ksq1

{State 1 - basal parameter values from Brewer et al 2016 [3]}
ken1=0.1218
kfe1 = 0.0345
kfg1=0.00047
kso1 = 0.0313
ksq1=0.0078
```

3. Script for simulation and integration of rate equations – Catalysis model

```
{integration set up}
d/dt (p) = krc*se + kf*gsv - p*ken
d/dt (se) = p*ken + krd*gsv - se*(krc+ksq*C)
d/dt (gsv) = se*ksq*C - (kf+krd)*gsv
C = Co - gsv

{the equations below can be used for analysis of antibody binding data}
d/dt (lp) = krc*ulse + kf*ulgsv
d/dt (ulse) = krd*ulgsv - ulse*(krc+ksq*C)
d/dt (ulgsv) = ulse*ksq*C - (kf+krd)*ulgsv

LIMIT p >= 0.0
LIMIT se  >= 0.0
LIMIT gsv >= 0.0

{Initial steady-state concentrations are based on algebraic solutions of the simultaneous
equations above and with the constrains p+gsv+se =1 and C=Co-gsv}
ROOTI q = q*q*ksq1*(kf1/ken1+1) -q*((kf1+krd1)*(krc1/ken1+1)+ksq1+ksq1*Co1*(kf1/ken1+1))
+ ksq1*Co1
Guess q = 0.5
LIMIT q <= 0.99
LIMIT q >=0.0001

INIT gsv = q
INIT se = (1- q*(kf1/ken1 + 1))/(krc1/ken1 + 1)
INIT p = 1-q-se

INIT ulgsv = q
INIT ulse = (1- q*(kf1/ken1 + 1))/(krc1/ken1 + 1)
INIT lp = 1-ulgsv-ulse

{State 2 - insulin rate constants in 3T3L1 were calculated by fitting the reference B to I transition
curve Fig. 1b derived from Brewer et al [3] parameters. Changing kf alone gives a good fit for
the Catalysis model}
ken = ken1
kf = 0.0368
ksq = ksq1
```

krc = krc1
krd = krd1
Co = Co1

{State 1 - basal rate constants in 3T3L1 were calculated by fitting to a reference antibody binding curve Fig. 1a (dashed red-line) derived from Brewer et al [3] parameters }
ken1= 0.13
kf1 = 0.0002
ksq1 = 0.433
krc1 = 0.014
krd1 = 0.0003
Co1 = 0.85
{Co1 is the partitioning factor that determines the fractional maximum level of GLUT4 in GSV in the basal state}

References for Supplement S1 a,b.

1 Coster, A. C., Govers, R. and James, D. E. (2004) Insulin stimulates the entry of GLUT4 into the endosomal recycling pathway by a quantal mechanism. Traffic. **5**, 763-771

2 Brewer, P. D., Habtemichael, E. N., Romenskaia, I., Mastick, C. C. and Coster, A. C. (2014) Insulin-regulated Glut4 translocation: membrane protein trafficking with six distinctive steps. J Biol Chem. **289**, 17280-17298

3 Brewer, P. D., Habtemichael, E. N., Romenskaia, I., Coster, A. C. and Mastick, C. C. (2016) Rab14 limits the sorting of Glut4 from endosomes into insulin-sensitive regulated secretory compartments in adipocytes. Biochem J. **473**, 1315-1327

4 Holman, G. D., Lo Leggio, L. and Cushman, S. W. (1994) Insulin-stimulated GLUT4 glucose transporter recycling. A problem in membrane protein subcellular trafficking through multiple pools. J Biol Chem. **269**, 17516-17524

5 Fazakerley, D. J., Holman, G. D., Marley, A., James, D. E., Stockli, J. and Coster, A. C. (2010) Kinetic evidence for unique regulation of GLUT4 trafficking by insulin and AMP-activated protein kinase activators in L6 myotubes. J Biol Chem. **285**, 1653-1660

6 Cornish-Bowden, A. (1976) 9 - Fast Reactions. In Principles of Enzyme Kinetics (Cornish-Bowden, A., ed.). pp. 153-167, Butterworth-Heinemann

7 Lizunov, V. A., Matsumoto, H., Zimmerberg, J., Cushman, S. W. and Frolov, V. A. (2005) Insulin stimulates the halting, tethering, and fusion of mobile GLUT4 vesicles in rat adipose cells. J Cell Biol. **169**, 481-489
